# Surgical site infection after wound closure with staples *versus* sutures in elective knee and hip arthroplasty: a systematic review and meta-analysis

**DOI:** 10.1186/s42836-021-00110-7

**Published:** 2022-03-04

**Authors:** A. van de Kuit, R. J. Krishnan, W. H. Mallee, L. M. Goedhart, B. Lambert, J. N. Doornberg, T. M. J. S. Vervest, J. Martin

**Affiliations:** 1 University of Groningen, University Medical Center Groningen, Groningen, The Netherlands; 2 Department of Anesthesia & Perioperative Medicine and Department of Epidemiology & Biostatistics, MEDICI Centre, University of Western Ontario, London, Canada; 3 Department of Orthopaedics, Joint Research, Onze Lieve Vrouwe Gasthuis, Amsterdam, the Netherlands; 4 Department of Orthopaedics, University Medical Center Groningen, Postbus 30.001, 9700 RB Groningen, The Netherlands; 5 Department of Orthopaedics, Tergooi Hospital, Hilversum, The Netherlands

**Keywords:** Surgical site infection, Wound closure, Total knee replacement, Total hip replacement, Arthroplasty, Systematic review

## Abstract

**Purpose:**

This systematic review and meta-analysis aimed to study surgical site infection of wound closure using staples versus sutures in elective knee and hip arthroplasties.

**Methods:**

A systematic literature review was performed to search for randomized controlled trials that compared surgical site infection after wound closure using staples *versus* sutures in elective knee and hip arthroplasties. The primary outcome was surgical site infection. The risk of bias was assessed with the Cochrane risk of bias assessment tool. The relative risk and 95% confidence interval with a random-effects model were assessed.

**Results:**

Eight studies were included in this study, including 2 studies with a low risk of bias, 4 studies having ‘some concerns’, and 2 studies with high risk of bias. Significant difference was not found in the risk of SSI for patients with staples *(n =* 557) *versus* sutures *(n =* 573) (RR: 1.70, 95% CI: 0.94–3.08, I^2^ = 16%). The results were similar after excluding the studies with a high risk of bias (RR: 1.67, 95% CI: 0.91–3.07, I^2^ = 32%). Analysis of studies with low risk of bias revealed a significantly higher risk of surgical site infection in patients with staples (*n* = 331) compared to sutures (*n* = 331) (RR: 2.56, 95% CI: 1.20–5.44, I^2^ = 0%). There was no difference between continuous and interrupted sutures (*P* > 0.05). In hip arthroplasty, stapling carried a significantly higher risk of surgical site infection than suturing (RR: 2.51, 95% CI: 1.15–5.50, I^2^ = 0%), but there was no significant difference in knee arthroplasty (RR: 0.87, 95% CI: 0.33–2.25, I^2^ = 22%; *P* > 0.05).

**Conclusions:**

Stapling might carry a higher risk of surgical site infection than suturing in elective knee and hip arthroplasties, especially in hip arthroplasty.

**Supplementary Information:**

The online version contains supplementary material available at 10.1186/s42836-021-00110-7.

## Background

In arthroplasty, surgical site infections (SSI) are an important problem associated with prosthetic joint infection, prolonged hospitalization, reoperation, readmission, increased mortality rates and increased healthcare costs [1, 2]. To prevent SSI, the World Health Organization (WHO) released the global guidelines (2016) for the use of antimicrobial-coated sutures [3]. However, the guidelines do not address the ongoing debate of stapling *versus* suturing in wound closure.

Owing to the low incidence of SSI, many randomized controlled trials (RCT) are underpowered and devaluated with a high risk of bias. Krishnan and colleagues indicated that patients in orthopedic and trauma surgery randomized to staples had a higher risk of SSI compared to patients randomized to sutures (RR, 2.05; 95% CI,1.38 to 3.06) [4]. However, there was no significant difference after excluding the studies with a high risk of bias [4]. Furthermore, both elective and trauma cases were included, causing heterogeneity in the study population because post-traumatic skin perfusion and wound healing are more difficult [5].

Recently, Mallee *et al.* [6] published the largest RCT in patients undergoing elective hip arthroplasty. This study showed a nearly three times greater risk of SSI after wound closure using staples *versus* sutures [6]. Given this recent evidence and the potential implications for SSI in patients undergoing arthroplasty in the elective setting, an analysis of available evidence is warranted. Therefore, we raised 4 questions: (1) Are staples or sutures associated with higher risk of SSI? (2) Do the results change when the analysis is limited to studies with a low risk of bias? (3) Do the outcomes differ between continuous and interrupted sutures? and (4) Do outcomes differ between hip and knee arthroplasties?

## Methods

We performed a systematic review and meta-analysis of RCT on SSI after primary hip and knee arthroplasties. We compared the risks of stapling *versus* suturing. The study was performed according to the Cochrane guidelines [7] and reported following the Preferred Reporting Items for Systematic Reviews and Meta-Analysis guidelines (PRISMA) guidelines [8].

### Primary outcome

Our primary outcome was SSI, defined as either superficial or deep infections. If the authors did not specify if infection is superficial or deep, we included their reported SSI. In case both categories were described, we assumed that we could combine the cases. When SSI was reported at multiple time points, the one with the longest available follow-up time was selected.

### Search strategy and study selection

We systematically searched the electronic databases PubMed, Embase, Cinahl, Cochrane, Google Scholar and Web of Science (Table S1 of the Appendix). Furthermore, we searched the first 15 pages of Google to identify potential studies that were not included in the databases mentioned above or had been published in non-indexed journals. Our final search was conducted on December 23, 2020. Then, two researchers (RK and AK) independently assessed the studies. Our inclusion criteria were: (1) RCTs; (2) adult patients (> 18 years of age); (3) elective knee and hip arthroplasties; (4) studies comparing stapling and suturing, and (5) reporting SSI as an outcome. The exclusion criteria were: (1) a barbed suture method because it was different from the methods mentioned above, (2) the use of synthetic adhesives such as 2-octyl cyanoacrylate; and (3) mixed populations (both trauma and elective surgery). A third researcher (JD) was available for resolving disputes about the eligibility of inclusion.

### Data extraction

One author (AK) extracted the data from the included studies. The second author (RK) verified the included data. We translated non-English trials using Google Translate. If there were questions about the (possibly) included studies, we personally communicated with the corresponding authors.

### Risk of bias

Using the Cochrane risk of bias assessment tool 2.0, we assessed the outcomes of the included studies to find an overall risk-of-bias score [9]. Algorithms were used to determine the risk of bias per domain and the overall risk of bias score. Three independent researchers (LG, BL and AK) assessed the risk of bias for included RCTs. Discrepancies were solved by comparing notes. A fourth researcher (JD) was available for resolving the disputes about biases.

### Statistical analysis

Statistical analysis was conducted using Review Manager (RevMan), version 5.4 (The Nordic Cochrane Centre, The Cochrane Collaboration, 2009, Copenhagen, Denmark) and Stata 15 (StataCorp LP, College Station, TX, USA). We calculated relative risk (RR) and 95% confidence interval (CI) with a random effects model [10]. We explored the publication bias using funnel plots and Egger’s regression test. Statistical heterogeneity was quantified by using the I^2^ value. A value equal to 40% or more was considered to be substantial heterogeneity [7]. Pre-planned subgroup analysis included a comparison between stapling and continuous and interrupted suturing in hip and knee arthroplasties. We used *post hoc* subgroup analyses to explore the effect size. Differences across subgroups were interpreted through the test for subgroup interaction, where a conservative threshold of *P* < 0.10 was used to indicate significant differences across subgroup effect sizes since tests for subgroup interactions are typically underpowered. Sensitivity analyses were performed to assess whether conclusions were affected when studies with “some concerns” or “high risk of bias” were excluded.

## Results

**Fig. 1:**
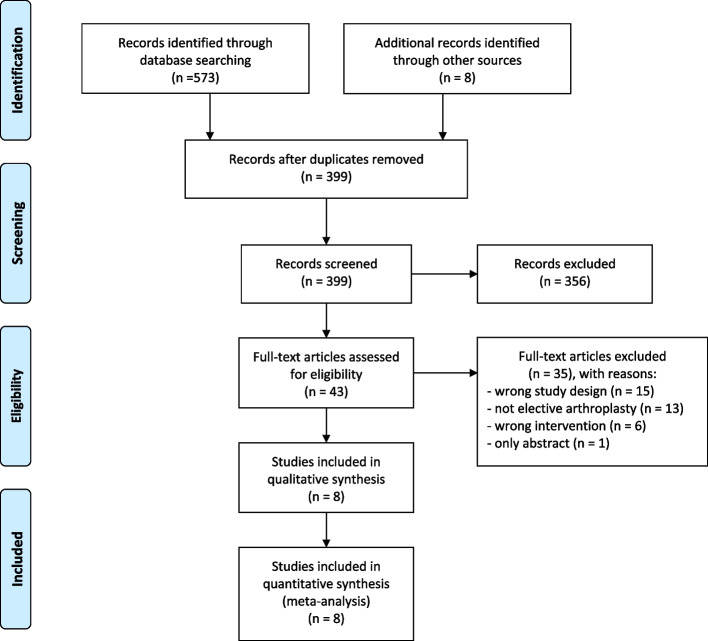
Flow of the identified studies

Our systematic search retrieved 399 unique studies (Fig. 1), and 356 of them were excluded based on title and abstract screening. After reading the full text, we excluded another 35 studies with erroneous study design or intervention (Table 2 of the Appendix). Finally, a total of 8 studies met the inclusion criteria (Table 1) [6, 11–17]. One of them was not available in English and was translated using Google Translate [14]. We extracted pre-defined study characteristics and the incidence of SSI in both groups.

**Table 1:** Study Characteristics

Study	*n*	Age	Female	Country	Intervention	Incidence SSI (Overall, staples vs. sutures(%))	Material of suturing	Type of suturing	Removal of staples (days)	Follow-up	Definition infection
Buttaro [11]	231	62^*^	52%	Argentina	Hip arthroplasty	0.4 (0.8 *vs*. 2.5)	Prolene 3–0	Continuous	15	45 days	Minor complications when medical treatment is required, and major complications when surgical treatment is required
Eggers [12]	38	68*	N.I.	USA	Knee arthroplasty	15.7 (5.2 *vs*. 26.3)	Monocryl 4–0	Continuous**	N.I.	6 weeks	N.I.
Graham [13]	20	57–82†	90%	UK	Knee arthroplasty	0	Vicryl 4–0	Continuous**	N.I.	7 days	N.I.
Hlubek [14]	72	69*	71%	Czech Republic	Knee arthroplasty	1.4 (2.6 *vs*. 0)	Ethilon 2–0	Interrupted	N.I.	6 weeks	N.I.
Khan [15]	127	70†	45%	Australia	Hip and Knee arthroplasty	7.0 (11.1 *vs*. 3.1)	Monocryl 3–0	Continuous	10	12 weeks	Positive culture or evidence of cellulitis
Mallee [6]	535	70*	33%	Netherlands	Hip arthroplasty	4.3 (6.0 vs. 2.6)	Absorbable and non-absorbable Ethilon	Continuous and Donati (interrupted)	14	One year	﻿Primary outcome SSI. At least 1 of the following: (1) purulent drainage, (2) organisms isolated (3) at least 1 of the signs or symptoms of infection, (4) diagnosis of superficial incisional SSI made by surgeon. Secondary outcome deep infection. At least 1 of the following: ﻿(1) purulent drainage from the deep incision; (2) a deep incision spontaneously dehisces or is deliberately opened by a surgeon when the patient has at least 1 of the following signs or symptoms: fever (> 38 °C), localized pain or tenderness, unless incision is culture-negative; (3) an abscess or other evidence of infection involving the deep incision is found on direct examination, during reoperation, or by histopathologic or radiologic examination; (4) diagnosis of deep incisional.
Nepal [17]	62	70*	82%	Thailand	Knee arthroplasty	0	Monocryl 3–0	Continuous	14	3 months	N.I.
Wyles [16]	45	70*	67%	USA	Knee arthroplasty	2.2 (0 vs. 3.3)	Monocryl 3–0	Continuous and Donati (interrupted)	14	3 months	N.I.

* Mean age, † Median or range, N.I. No information

** The study did not indicate whether interrupted or continuous sutures were used. Based on the use of the absorbable suturing material, we interpreted that the suturing was continuous

### Study characteristics

In the 8 RCTs, 5 studies focused on knee arthroplasty [12–14, 16, 17], 2 studies on hip arthroplasty [6, 11], and 1 study on both hip and knee arthroplasty [15]. A total of 1130 patients (stapling in 557 patients and suturing in 573 patients) were included in the meta-analysis. In 2 studies, SSI was the primary outcome [6, 15]. In the remaining 6 studies, SSI was described as one of the secondary outcomes. The incidence of SSI varied from 0 to 15.7%. We did not observe wound complications or SSI in 2 studies [13, 17]. A clear description of SSI was reported in 2 studies [6, 15]. The remaining 6 studies did not provide a clear definition of SSI. Absorbable sutures were used in 5 studies and non-absorbable sutures were used in 3 studies [6, 11, 16]. The follow-up period ranged from 7 days to 1 year.

### Risk of Bias

Two studies had low risk of bias [6, 15], 4 studies had ‘some concerns’ [11, 12, 16, 17], and 2 studies had high risk of bias [13, 14]. Owing to the intervention nature, blinding of patients and outcome assessors was impossible in all studies. In 2 studies, the authors tried to prevent bias by blinding the outcome assessor or statistician [6, 15]. In 1 study, the follow-up lasted for 7 days, which was insufficient for detecting SSI [13]. The important reasons for being rated as high risk were the concerns about SSI detected (Appendix Table 3 and 4). In many studies [11–14, 16, 17], there was lack of description of SSI, which influenced the intervention. Other concerns included the lack of information about the randomization process or predictable allocation sequence (quasi-randomization) (Table 2).

**Table 2:**
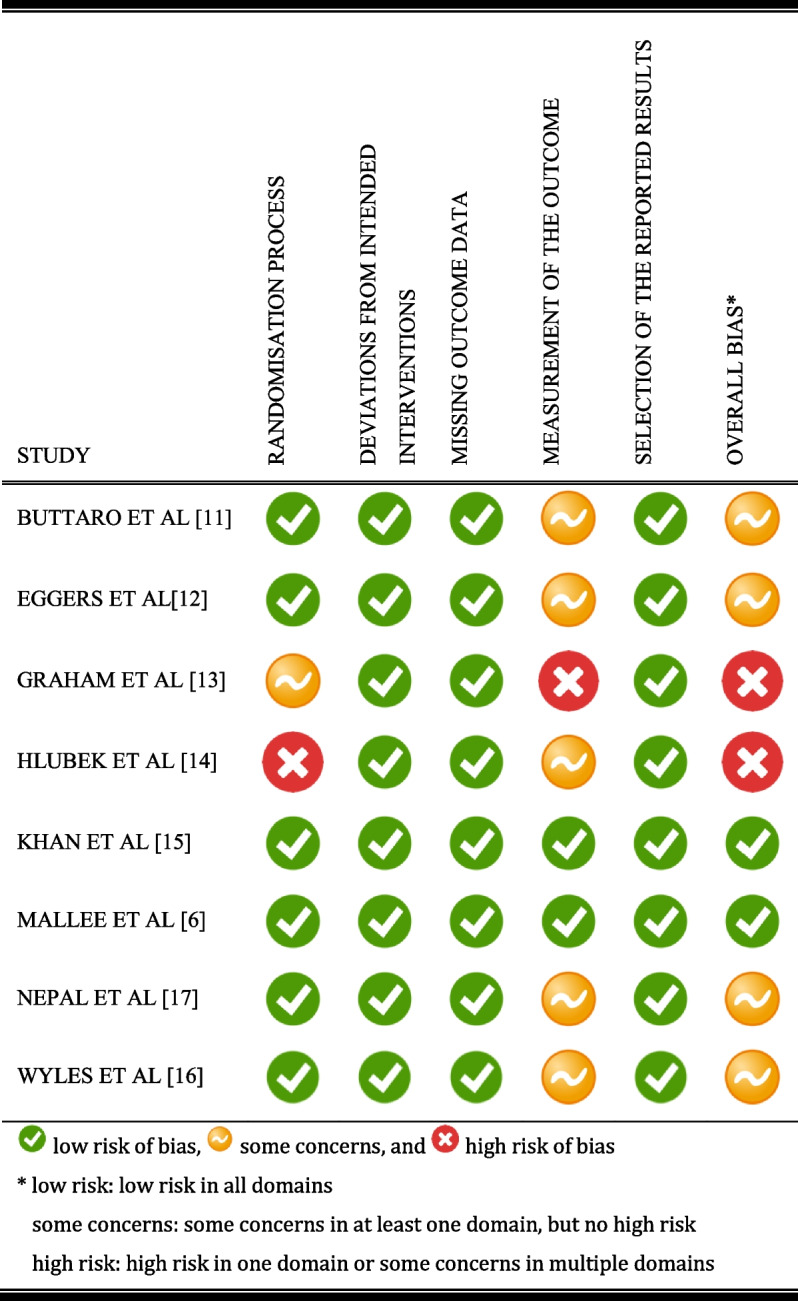
Risk of Bias in the included studies

### Meta-analysis

**Fig. 2:**
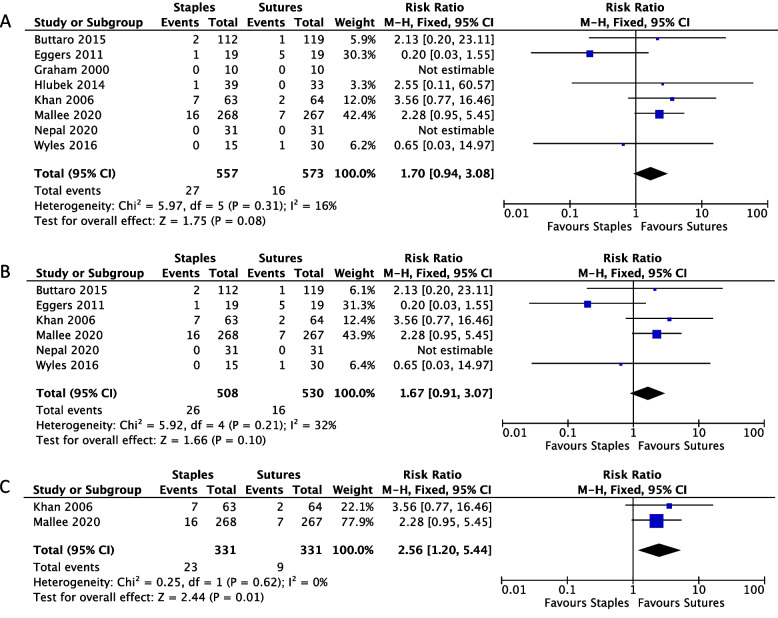
Forest plot showing the relative risk (RR, 95% CI) for patients receiving staples *versus* sutures for wound closure in arthroplasty in (**A**) all included RCTs, (**B**) RCTs with some concerns and low risk of bias, and (**C**) RCTs with only low risk of bias

In all RCTs combined, we did not find a significant difference in the risks of SSI between suturing (*n* = 557) and stapling *(n =* 573) (RR: 1.70, 95% CI: 0.94–3.08, I^2^ = 16%) (Fig. 2A). The results were similar after excluding the studies with high risk of bias, where we did not find a significant difference between stapling *(n =* 508) and suturing (*n* = 530) (RR: 1.67, 95% CI: 0.91–3.07, I^2^ = 32%) (Fig. 2B). When the analysis was limited to the studies with a low risk of bias (Mallee *et al*. and Khan* et al*.), we found a significantly higher risk of SSI for patients treated with staples (*n* = 331) compared to sutures (*n* = 331) (RR: 2.56, 95% CI: 1.20–5.44, I^2^ = 0%) (Fig. 2C).

Subgroup analyses did not suggest significant differences in the risks of SSI between staples and interrupted sutures and between staples and continuous sutures (*P* value for subgroup interaction = 0.99) (Fig. S1). For the hip arthroplasty subgroup, staples were associated with an increased risk of SSI compared to sutures (RR: 2.51, 95% CI: 1.15–5.50, I^2^ = 0%), but there was no difference in knee arthroplasty (RR:0.87, 95% CI: 0.33–2.25, I^2^ = 22%). The test for interaction across subgroups indicated a significant difference in effect size between the two subgroups (*P* value for subgroup interaction = 0.09) (Fig. S2). Statistical analysis of publication bias was not feasible because the number of our studies was smaller than 10 [7].

## Discussion

In this systematic review and meta-analysis, we assessed the risk of SSI after primary wound closure with staples *versus* sutures in elective knee and hip arthroplasty. Our primary findings suggest that wound closure with staples carries a higher risk of SSI than sutures. Furthermore, we showed that the subgroup of patients undergoing hip arthroplasty may have a higher risk of SSI when treated with staples, but no difference was found for knee arthroplasty. However, given the low power in this meta-analysis and the heterogeneity of SSI definitions, the results remain non-definitive.

This is the first meta-analysis that focused primarily on wound closure with staples and sutures in elective hip and knee arthroplasties. Previous reviews focused on heterogeneous groups, including both trauma as well as elective patients, or focused on other closing materials such as barbed sutures [4, 18–20]. The strength of our study includes a large number of patients (1130 patients), a relatively sufficient number of RCTs (8 studies), and study of elective arthroplasties. Using the Cochrane risk of bias assessment tool 2.0, we assessed, in detail, the risks of bias based on the 5 different domains, and conducted a sensitivity analysis by excluding the studies with a higher risk of bias.

The major limitation of this systematic review is the lack of high-quality and adequately-powered studies in arthroplasty. Future studies should primarily focus on SSI, more clearly describe the outcomes (*e*.*g*., definitive data from Centers for Disease Control and Prevention and at least 1 year follow-up to detect all potentially serious SSI), and have definitive results based on larger sample size. Second, double blinding was largely absent in the included studies due to the nature of the interventions but should be possible for the statistician in future studies. Third, the heterogeneous population (including both knee and hip arthroplasties) and different postoperative wound managements and healing processes decrease the power of the meta-analysis, resulting in a non-definitive conclusion [5].

Joint arthroplasty remains one of the most common surgical procedures and is a significant contributor to SSI burden worldwide. It is crucial to identify the interventions to reduce the risk of SSI. When choosing a wound closure method, we should consider these factors, including availability, familiarity, affordability, cost-effectiveness, cosmetic outcome, and patient and surgeon preferences. Stapling does reduce surgical time, but is a more expensive option [17, 21, 22].

Krishnan *et al*. [4] performed a meta-analysis and found that stapling had a higher incidence of SSI than suturing in elective and traumatic arthroplasties, but the inclusion was not limited to studies with a low risk of bias. It is unclear whether the SSI is associated with soft tissue reaction to stainless steel and titanium of staples, wound tension, the lack of perfusion, or poor techniques [21]. Overlapping or inverted wound edges may cause persistent oozing and infection at skin entry points [5, 23]. Several studies provided a description of stapling techniques [12, 13, 17]. One of them showed that wound oxygenation is similar in skin closure when subcuticular Vicryl or staples were used [13]. Theoretically, greater space between staples may provide an advantage in terms of oxygenation. Therefore, during wound closure, (1) the assistant should help prevent overlapping or inversion of the wound edges using toothed forceps, and (2) ensure enough spacing between staples (at least 6 mm) [5, 13].

No difference between subgroups was found for continuous and interrupted sutures; however, the subgroup analysis was underpowered. Liu* et al*. [24] studied the differences between running absorbable and vertical mattress nonabsorbable sutures in total knee arthroplasty, and also showed no difference in infection rates. We therefore hypothesize that the suturing type may be less relevant than the ongoing debate between stapling *versus* suturing.

In hip arthroplasty, we found stapling was associated with an increased risk of SSI, compared to suturing. The underlying mechanism remains unclear but possibly involves a longer incision in knee arthroplasty, associated with more mobility than hip arthroplasty [15, 20]. Suturing may be a preferential option to stapling in hip arthroplasty*.*

## Conclusion

Stapling might carry a higher risk of surgical site infection than suturing in elective knee and hip arthroplasties, especially in hip arthroplasty.

## Supplementary Information

**Additional file 1.**

## Data Availability

No additional data are available.

## Abbreviations

SSI: Surgical Site Infection
RR: Relative Risk
CI: Confidence Interval
WHO: World Health Organization
RCT: Randomized Controlled Trial
