# Supplementary material for: Surgical site infection after wound closure with staples versus sutures in elective knee and hip arthroplasty: a systematic review and meta-analysis
Source: Arthroplasty. 2022 Mar 4;4:12. doi: 10.1186/s42836-021-00110-7 (PMC8896293; doi:10.1186/s42836-021-00110-7)
Supplement: Supplementary file 1 — Additional file 1. [file 42836_2021_110_MOESM1_ESM.docx]

**Supplementary Appendix**

Table S1. Predefined search strategy.

| **Embase** | ('orthopedic surgery'/exp OR 'orthopedic surgery' OR 'orthopaedic surger*' OR ‘trauma surg*’ OR 'traumatology'/exp OR 'arthroplast*' OR 'knee replacement*' OR 'joint replacement*' OR 'hip replacement*' OR 'knee arthroplast*' OR 'hip arthroplas*' OR 'joint arthroplast*' OR 'hemiarthroplasty') AND ('suture technique'/exp OR 'suture technic*' OR 'suture technique*' OR 'surgical sutur*' OR 'suture*' OR ‘vicryl’) AND ('skin clip*' OR 'stapl*' OR 'skin stapl*' OR 'surgical staple'/exp OR 'surgical stapl*') AND ('wound healing'/exp OR 'wound healing' OR 'wound closure'/exp OR 'wound closure' OR 'cicatri*' OR 're-epithelializ*' OR 'skin closur*' OR 'wound infection'/exp) |
| --- | --- |
| **PubMed** | ("orthopedics"[MeSH Terms] OR "orthopedic surger*" OR "orthopaedic surger*" OR "traumatology"[MeSH Terms] OR "trauma surg*" OR "fracture surg*" OR "orthopedic" OR "arthroplast*" OR "knee replacement*" OR "joint replacement*" OR "hip replacement*" OR "knee arthroplast*" OR "hip surg*" OR "joint surg*") AND ("wound closure techniques"[MeSH Terms] OR "suture technic*" OR "suture technique*" OR "surgical sutur*" OR "sutur*" OR "vicryl") AND ("surgical staplers"[MeSH Terms] OR "staple*" OR "surgical stapl*" OR "skin clip*") AND ("wound healing" OR "wound compl*" OR "wound closure" OR "cicatri*" OR "re-epithelializ*" OR "skin closur*" OR "SSI" OR "infection*" OR "wound healing"[MeSH Terms] OR "wound healing/complications"[MeSH Terms]) |
| **Cinahl** | ("orthopedic surger*" OR "orthopaedic surger*" OR "arthroplast*" OR "knee replacement*" OR "joint replacement*" OR "hip replacement*" OR "knee arthroplast*" OR "hip arthroplas*" OR "joint arthroplast*") AND ("suture technic*" OR "suture technique*" OR "surgical stapl*" OR "surgical sutur*" OR "skin clip*" OR "suture*" OR "staple*") AND ("wound healing" OR "wound closure" OR "cicatri*" OR "re-epithelializ*" OR "skin closur*") |
| **Cochrane** | ("orthopedic surger*" OR "orthopaedic surger*" OR "arthroplast*" OR "knee replacement*" OR "joint replacement*" OR "hip replacement*" OR "knee arthroplast*" OR "hip arthroplas*" OR "joint arthroplast*") AND ("suture technic*" OR "suture technique*" OR "surgical stapl*" OR "surgical sutur*" OR "skin clip*" OR "suture*" OR "staple*") AND ("wound healing" OR "wound closure" OR "cicatri*" OR "re-epithelializ*" OR "skin closur*") |
| **Web of Science** | ("orthopedic surger*" OR "orthopaedic surger*" OR "arthroplast*" OR "knee replacement*" OR "joint replacement*" OR "hip replacement*" OR "knee arthroplast*" OR "hip arthroplas*" OR "joint arthroplast*") AND ("suture technic*" OR "suture technique*" OR "surgical stapl*" OR "surgical sutur*" OR "skin clip*" OR "suture*" OR "staple*") AND ("wound healing" OR "wound closure" OR "cicatri*" OR "re-epithelializ*" OR "skin closur*") |
| **Google** | Staples, sutures, orthopedic surgery, arthroplasty |

| **Date searched: December 23, 2020** | **Total studies captured** |
| --- | --- |
| Pubmed (Medline) | 108 |
| Embase | 178 |
| Cinahl | 120 |
| Cochrane | 37 |
| Web of Science | 130 |
| Google | 8 |
| **Total** | **573** |

Table S2. Studies excluded after reading full text.

| Study | Reason for exclusion |
| --- | --- |
| Ali Shah F, Amjad Ali M, Zia Khan U. Surgical site wound complication; a comparative study of surgical site wound complication rates of metallic skin staples versus polypropylene sutures in orthopaedic wound closure. *Prof Med J*. 2018;25(10):1487-1491. | - Intertrochantic fractures. |
| Almustafa MA, Ewen AM, Deakin AH, Picard F, Clarke J V, Mahmood FF. Risk Factors for Surgical Site Infection Following Lower Limb Arthroplasty: A Retrospective Cohort Analysis of 3932 Lower Limb Arthroplasty Procedures in a High Volume Arthroplasty Unit. *J Arthroplasty*. 2018;33(6):1861-1867. | - Retrospective design |
| Badres IA, Suen K, Tran P. Effect of Wound Closure Technique in Proximal Femoral Fractures: A Prospective Cohort Study. *J Orthop Trauma*. 2020;34(10):553-558. | - Trauma surgery |
| Campbell AL, Patrick DA, Liabaud B, Geller JA. Superficial Wound Closure Complications with Barbed Sutures Following Knee Arthroplasty. *J Arthroplasty*. 2014;29(5):966-969. | - Barbed sutures versus staples |
| Chaudhary P, Shrestha BP, Khanal GK, Rijal R, Maharjan R. Randomized controlled trial comparing outcome of use of staples and nylon sutures for closure in elective orthopedic surgery. *Heal Renaiss*. 2017;13(3):137-143. | - Mixed population and not separately reported |
| Chawla H, van der List JP, Fein NB, Henry MW, Pearle AD. Barbed Suture Is Associated With Increased Risk of Wound Infection After Unicompartmental Knee Arthroplasty. *J Arthroplasty*. 2016;31(7):1561-1567. | - Retrospective design  - Barbed sutures |
| Clayer M, Southwood RT. Comparative study of skin closure in hip surgery. *ANZ J Surg*. 1991;61(5):363-365. | - Mixed population and not seperately reported |
| Daniilidis K, Stukenborg-Colsman C, Ettinger S, et al. Nylon sutures versus skin staples in foot and ankle surgery: is there a clinical difference? *Musculoskelet Surg*. 2020;104(2):163-169. | - Retrospective design |
| Glennie RA, Korczak A, Naudie DD, Bryant DM, Howard JL. MONOCRYL and DERMABOND vs Staples in Total Hip Arthroplasty Performed Through a Lateral Skin Incision: A Randomized Controlled Trial Using a Patient-Centered Assessment Tool. *J Arthroplasty*. 2017;32(8):2431-2435. | - Sutures AND glue  - No SSI as outcome |
| Gohiya DA, Gupta DDC, Gaur DPS. Comparative Study of Outcome of two Methods (Sutures Vs Staples) of Skin Closure in Orthopedic Surgery. *Int J Med Res Rev*. 2015;3(1):16-22. | - Mixed population |
| Hettwer WH, Horstmann PF, Wu C, Petersen MM. Comparison of two alternative wound closure methods for tumor arthroplasty of the hip: A frequency matched cohort study. *J Orthop Surg*. 2018;26(3). | - Patients had tumor resection or pathologic fracture  - Sutures with glue and steri-strips |
| S. L. Karlakki, A. K. Hamad, C. Whittall, N. M. Graham, R. D. Banerjee, J. H. Kuiper. Incisional negative pressure wound therapy dressings (iNPWTd) in routine primary hip and knee arthroplasties: A randomised controlled trial. Bone Joint Res 2016;5:328-337. | - Groups used also negative pressure wound therapy. |
| Kazemian G, Manafi Rasi A, Tavakoli Darestani R, Reza Ebrahiminia M, Khani S, Safdari F. Comparison of Suture and Staple in Closing the Wounds of Surgery of Intertrochanteric Fractures. *J Appl Environ Biol Sci*. 2014;4(3s):112-115. | - Intertrochantic fractures |
| Lehtonen E, Patel H, Phillips S, Correia Pinto M, Naranje S, Shah A. Staple versus suture closure for ankle fracture fixation: Retrospective chart review for safety and outcomes. *Foot*. 2018;37:71-76. | - Traumatic ankle fractures |
| Liew SM, Haw CS. The use of taped skin closure in orthopaedic wounds. *Aust N Z J Surg*. 1993;63(2):131-133. | - Type of surgery not specified |
| Lu Y, Wang C, Lin L, Qin Q, Li Q. Complication rate of different wound closures after primary hip arthroplasty - A survey of 373 patients. *Asia Pac J Sport Med Arthrosc Rehabil Technol*. 2018;11:15-18. | - Retrospective design |
| Moore DC, Sellers MH, Archer KR, Schwartz HS, Holt GE. Staples Equal Sutures for Skin Closure After Soft Tissue Tumor Resection. *Clin Orthop Relat Res*. 2013;471(3):899-904. | - Retrospective design  - Resection of sarcoma |
| Murphy M, Prendergast P, Rice J. Comparison of clips versus sutures in orthopaedic wound closure. *Eur J Orthop Surg Traumatol*. 2004;14(1):16-18. | - Fracture fixation also included, not separately reported |
| Newman JT, Morgan SJ, Resende G V, Williams AE, Hammerberg EM, Dayton MR. Modality of wound closure after total knee replacement: are staples as safe as sutures? A retrospective study of 181 patients. *Patient Saf Surg*. 2011;5(1):26. | - Retrospective design |
| Patel RM, Cayo M, Patel A, Albarillo M, Puri L. Wound complications in joint arthroplasty: comparing traditional and modern methods of skin closure. *Orthopedics*. 2012;35(5):e641-6. | - Retrospective design |
| Prabhakar G, Bullock TS, Martin CW, et al. Skin closure with surgical staples in ankle fractures: a safe and reliable method. *Int Orthop*. 2020. | - Ankle fractures  - Retrospective design |
| Roumeliotis L, Graham NM. Barbed suture and glue in skin closure during lower limb arthroplasty: reduced delayed discharge due to wound exudate. *J Wound Care*. 2019;28(11):784-789. | - Retrospective design  - No SSI data |
| Rui M, Zheng X, Sun SS, et al. A prospective randomised comparison of 2 skin closure techniques in primary total hip arthroplasty surgery. *HIP Int*. 2018;28(1):101-105. | - Mixed population |
| Shani A, Poliansky V, Mulla H, Rahamimov N. Nylon Skin Sutures Carry a Lower Risk of Post-Operative Infection than Metal Staples in Open Posterior Spine Surgery: A Retrospective Case-Control Study of 270 Patients. *Surg Infect (Larchmt)*. 2020;21(5):440-444. | - Retrospective design |
| Shetty AA, Kumar VS, Morgan-Hough C, Georgeu GA, James KD, Nicholl JE. Comparing wound complication rates following closure of hip wounds with metallic skin staples or subcuticular vicryl suture: a prospective randomised trial. *J Orthop Surg (Hong Kong)*. 2004;12(2):191-193. | - Proximal femur fractures |
| Singh H, Arora RK, Khanna G, Agrawal DK, Bansal B, Agrawal D. Study to detemine the best surgical skin wound closure material out of nylon, silk or staple, for use in various orthopedic surgical procedures. *IJOTSS*. 2017;3(1):426-434. | - Mixed population |
| Singhal AK, Hussain A. Skin closure with automatic stapling in total hip and knee arthroplasty. *JK Pract*. 2006;13(3):142-143. | - Retrospective design |
| Slade Shantz JA, Vernon J, Morshed S, Leiter J, Stranges G. Sutures versus staples for wound closure in orthopaedic surgery: A pilot randomized controlled trial. *Patient Saf Surg*. 2013;7(1):6. | - Mixed population, not separately described. |
| Stockley I, Elson RA. Skin closure using staples and Nylon sutures: A comparison of results. *Ann R Coll Surg Engl*. 1987;69(2):76-78. | - Mixed population, not separately described. |
| Taheriazam A, Safdari F. Comparison of sutures versus staples  in surgical wound closure after total hip replacement. *SICOT*  *40th Orthop world Congr*. 2019. | - Full text not available (after contacting corresponding author) |
| Uçkay I, Agostinho A Fau - Belaieff W, Belaieff W Fau - Toutous-Trellu L, et al. Noninfectious wound complications in clean surgery: epidemiology, risk factors, and association with antibiotic use. *World J Surg*. 35(5):973-980. | - Prospective observational study  - Mixed population |
| Vala P, Devda A, Vardey M, Goyal R, Upadhyay A. Wound Closure In Orthopaedics Surgery : Sutures versus Staples Dr Pathik Vala Dr Ajay Devda Dr Mehul Vardey Dr Rakesh Goyal Dr Arpit Upadhyay. *IJAR*. 2016;6(11):50-52. | - Mixed population |
| Wang L-S, Wang X-Y, Tu H, Huang Y-F, Qi X, Gao Y-H. Octyl-2-cyanoacrylate tissue adhesive without subcuticular suture for wound closure after total hip arthroplasty: a prospective observational study on thirty-two cases with controls for 3 months follow-up. *J Orthop Surg Res*. 2020;15(1):1-6. | - Sutures AND glue |
| Yuenyongviwat V, Iamthanaporn K, Hongnaparak T, Tangtrakulwanich B. A randomised controlled trial comparing skin closure in total knee arthroplasty in the same knee: nylon sutures versus skin staples. *Bone Joint Res*. 2016;5(5):185-190. | - Not a clear description of wound infection. |
| Zhu M, Rahardja R, Munro J, Coleman B, Young SW. Wound closure and follow-up after total knee arthroplasty - Do they affect the rate of antibiotic prescription? *Knee*. 2019;26(3):700-707. | - Retrospective design |


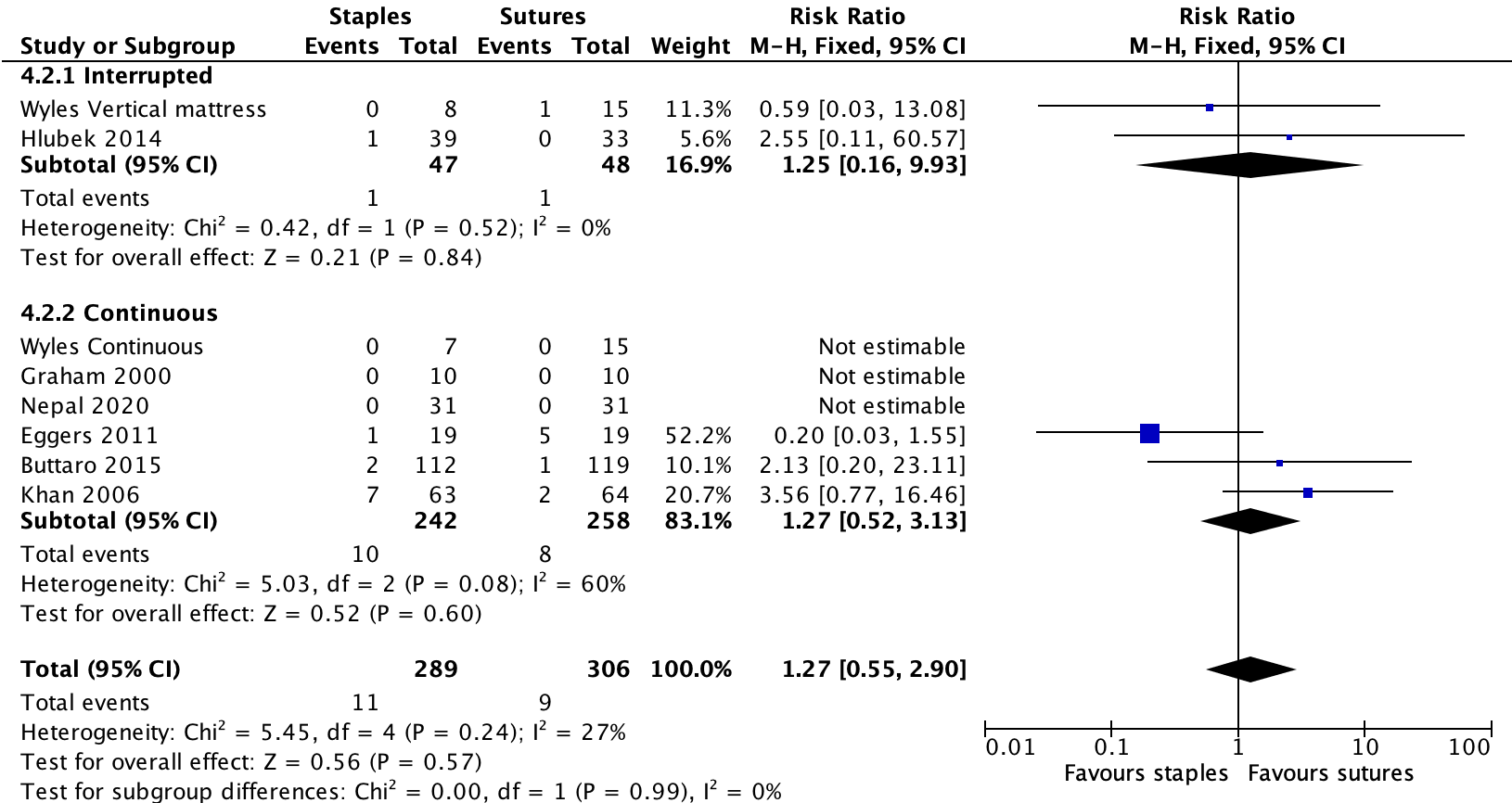


**Figure S1:** Forest plot showing the relative risk (RR, 95% CI) of SSI for patients receiving staples versus sutures for skin closure after elective arthroplasty, subgrouped by suturing method (interrupted vs continuous). In both interrupted and continuous suturing, we showed no difference in the risk of SSI compared with staples (respectively: RR: 1.25, 95% CI: 0.16-9.93, I^2^ = 0% and RR: 1.27, 95% CI: 0.52-3.13, I^2^ = 60%). The test for subgroup differences across the subgroups was not significant (p = 0.99).


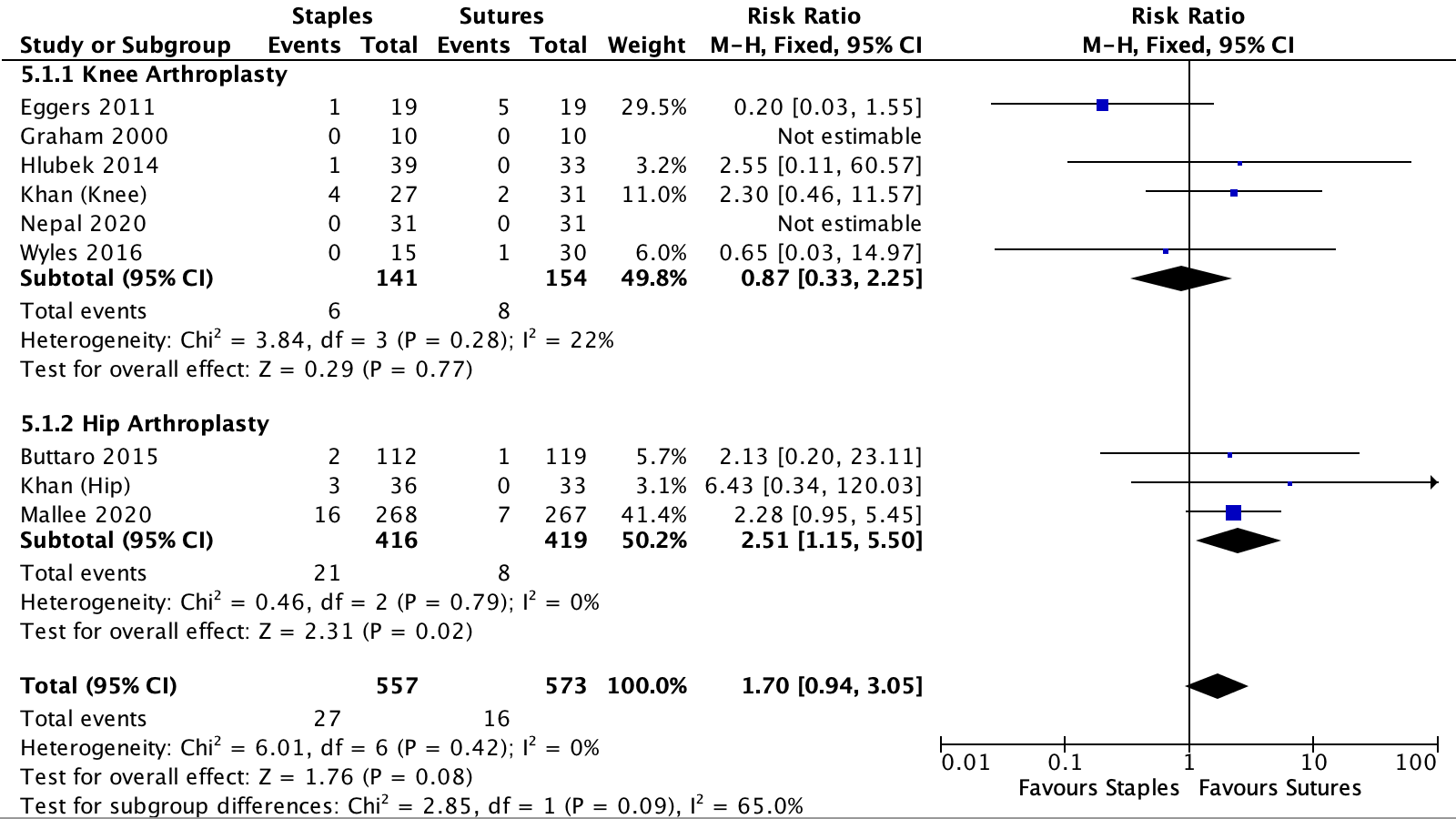


**Figure S2:** Forest plot showing the relative risk (RR, 95% CI) of SSI for patients receiving staples versus sutures for skin closure after elective arthroplasty, subgrouped by type of arthroplasty (knee vs. hip). In knee arthroplasty, no difference was found in the risk of SSI compared with staples (RR:0.87, 95% CI: 0.33-2.25, I^2^ = 22%). In hip arthroplasty, patients treated with staples had an increased risk of SSI (RR:2.51, 95% CI: 1.15-5.50, I^2^ = 0%). The test for subgroup differences across the subgroups indicated significant difference in effect size for these subgroups (p = 0.09).

Table S3. Comments on randomization process and measurement of outcome.

| Study | Randomization process | Measurement of the outcome | Comments |
| --- | --- | --- | --- |
| Buttaro | Low | Some concerns | No clear description of SSI: “﻿These complications were defined as minor, when they required medical treatment, and major complications, when they required surgical treatment.” |
| Eggers | Low | Some concerns | No clear description of SSI: “Secondary end points including … infection, …” |
| Graham | Some concerns | High | Randomization indicated, but not described: “﻿were randomly allocated to”. No definition of SSI at all. Only seven days follow-up. |
| Hlubek | High | Some concerns | Randomization based on date of birth. No clear definition of SSI: “The surgical wounds were subsequently monitored and evaluated by a single attending physician” (translated) |
| Khan | Low | Low | Randomly allocated and clear definition of SSI. |
| Mallee | Low | Low | Randomly allocated and clear definition of SSI. |
| Nepal | Low | Some concerns | No clear description of SSI: ﻿“specific wound com- plications that were monitored included … wound infection”. |
| Wyles | Low | Some concerns | No clear description of SSI: “﻿The incision site was checked for any complications at all followup appointments through a minimum of 3 months after surgery” |

Table S4. Comments on deviations on intended interventions, missing outcome data and selection of the reported results

| Study | Deviations of intended interventions | Missing outcome data | Selection of the reported results | Comments |
| --- | --- | --- | --- | --- |
| Buttaro | Low | Low | Low | Deviation not applicable, no missing outcome data and no selection of results. |
| Eggers | Low | Low | Low | Deviation not applicable, no missing outcome data and no selection of results. |
| Graham | Low | Low | Low | Deviation not applicable, no missing outcome data and no selection of results. |
| Hlubek | Low | Low | Low | Deviation not applicable, no missing outcome data and no selection of results. |
| Khan | Low | Low | Low | Deviation not applicable, no missing outcome data and no selection of results. |
| Mallee | Low | Low | Low | Deviation not applicable, no missing outcome data and no selection of results. |
| Nepal | Low | Low | Low | Deviation not applicable, no missing outcome data and no selection of results. |
| Wyles | Low | Low | Low | Deviation not applicable, no missing outcome data and no selection of results. |
